# Genome Sequence of the Facultative Anaerobe *Oerskovia enterophila* DFA-19 (DSM 43852^T^)

**DOI:** 10.1128/genomeA.00973-16

**Published:** 2016-09-15

**Authors:** Vanessa Jag, Anja Poehlein, Frank R. Bengelsdorf, Rolf Daniel, Peter Dürre

**Affiliations:** a Institut für Mikrobiologie und Biotechnologie, Universität Ulm, Ulm, Germany; b Genomic and Applied Microbiology and Göttingen Genomics Laboratory, Institute of Microbiology and Genetics, Georg-August Universität Göttingen, Göttingen, Germany

## Abstract

Here, we report the draft genome sequence of *Oerskovia enterophila* DFA-19 (DSM 43852^T^), a facultative anaerobe soil bacterium, which was originally isolated from millipede feces and first described as *Promicromonospora enterophila*. The genome consists of a circular chromosome comprising approximately 4.65 Mb and 4,044 predicted protein-encoding genes.

## GENOME ANNOUNCEMENT

The genus *Oerskovia* was first characterized in 1970 by Prauser et al. (1), and members are described as Gram-positive bacteria that show extensively branching vegetative hyphae, growing on and into the agar. In addition, a very variable cell shape was observed (1). The species *O. enterophila* was first described as *Promicromonospora enterophila* due to microscopic structures erroneously identified as spores and the supposed close relationship to the type strain *Promicromonospora citrae* (2). Subsequent phylogenetic analysis revealed *P. enterophila* as a member of the genus *Oerskovia*. Moreover, the type strain of *O. turbata* shows 99.6% 16S rRNA gene sequence similarity with the type strain of *P. enterophila*, and thus both are close phylogenetic neighbors (3).

For genome sequencing of *O. enterophila* DFA-19, the strain was received from the Deutsche Sammlung von Mikroorganismen und Zellkulturen (DSMZ) and was inoculated in 5 ml trypticase soy-yeast extract medium (medium 92; DSMZ) and incubated overnight at 30°C. Whole-genomic DNA was isolated using the MasterPure Gram-positive DNA purification kit (Epicentre, Madison, WI, USA). Isolated DNA was used to generate Illumina shotgun sequencing libraries. Sequencing was performed by employing a MiSeq system using a MiSeq reagent kit version 3 (600 cycles), as recommended by the manufacturer (Illumina, San Diego, CA, USA). Sequencing resulted in 2,498,636 paired-end reads that were trimmed using Trimmomatic version 0.32 (4). *De novo* assembly performed with the SPAdes genome assembler software version 3.8.0 (5) yielded 114 contigs (>500 bp) with an average coverage of 90.5-fold. The genome of *O. enterophila* DFA-19 (DSM 43852^T^) consists of a circular chromosome of 4.65 Mb with an overall G+C content of 72.28%. Gene prediction and annotation were performed using the Prokka rapid prokaryotic genome annotation tool (6). The genome harbored three rRNA genes, 58 tRNA genes, 2,820 protein-encoding genes with predicted functions, and 1,224 genes coding for hypothetical proteins.

Analysis of the genome revealed that *O. enterophila* DFA-19 has several genes encoding enzymes involved in the degradation of plant polymers that are ubiquitously available in soils, such as cellulose, starch, or chitin (7–9). Accordingly, *O. enterophila* DFA-19 was first isolated from millipede feces and described as a soil bacterium (2). Potential genes encoding two endoglucanases and one cellulose 1,4-beta-cellobiosidase are present in the *O. enterophila* DFA-19 genome. Furthermore, putative genes encoding amylases for the conversion of starch or glycogen to dextrin were identified. Additionally, genes encoding chitinases, chitin-binding proteins, and a chitobiose phosphorylase were also present in the genome.

### Accession number(s).

This whole-genome shotgun project has been deposited at DDBJ/ENA/GenBank under the accession number MAQA00000000. The version described in this paper is the first version, MAQA01000000.

## References

[1] Prauser H, Lechevalier MP, Lechevalier H 1970 Description of *Oerskovia* gen. n. to harbor Ørskov’s motile *Nocardia*. Appl Microbiol 19:534. 5440174 10.1128/am.19.3.534-534.1970 PMC376723

[2] Jáger K, Marialigeti K, Hauck M, Barabás G 1983 *Promicromonospora enterophila* sp. nov., a new species of monospore actinomycetes. Int J Syst Bacteriol 33:525–531. doi:10.1099/00207713-33-3-525.

[3] Stackebrandt E, Breymann S, Steiner U, Prauser H, Weiss N, Schumann P 2002 Re-evaluation of the status of genus *Oerskovia*, reclassification of *Promicromonospora enterophila* (Jáger et al. 1983) as *Oerskovia enterophila* comb. nov. and description of *Oerskovia jenensis* sp. nov. and *Oerskovia paurometabola* sp. nov. Int J Syst Evol Microbiol 52:1105–1111. doi:10.1099/00207713-52-4-1105. 12148614

[4] Bolger AM, Lohse M, Usadel B 2014 Trimmomatic: a flexible trimmer for Illumina sequence data. Bioinformatics 30:2114–2120. doi:10.1093/bioinformatics/btu170. 24695404 PMC4103590

[5] Bankevich A, Nurk S, Antipov D, Gurevich AA, Dvorkin M, Kulikov AS, Lesin VM, Nikolenko SI, Pham S, Prjibelski AD, Pyshkin AV, Sirotkin AV, Vyahhi N, Tesler G, Alekseyev MA, Pevzner PA 2012 SPAdes: a new genome assembly algorithm and its applications to single-cell sequencing. J Comput Biol 19:455–477. doi:10.1089/cmb.2012.0021. 22506599 PMC3342519

[6] Seemann T 2014 Prokka: rapid prokaryotic genome annotation. Bioinformatics 30:2068–2069. doi:10.1093/bioinformatics/btu153. 24642063

[7] Leschine SB 1995 Cellulose degradation in anaerobic environments. Annu Rev Microbiol 49:399–426. doi:10.1146/annurev.mi.49.100195.002151. 8561466

[8] Britz SJ, Hungerford WE, Lee DR 1987 Rhythms during extended dark periods determine rates of net photosynthesis and accumulation of starch and soluble sugars in subsequent light periods in leaves of *Sorghum*. Planta 171:339–345. doi:10.1007/BF00398679. 24227433

[9] Reguera G, Leschine SB 2001 Chitin degradation by cellulolytic anaerobes and facultative aerobes from soils and sediments. FEMS Microbiol Lett 204:367–374. doi:10.1111/j.1574-6968.2001.tb10912.x. 11731150

